# Enhanced Broad-Learning-Based Dangerous Driving Action Recognition on Skeletal Data for Driver Monitoring Systems

**DOI:** 10.3390/s25061769

**Published:** 2025-03-12

**Authors:** Pu Li, Ziye Liu, Hangguan Shan, Chen Chen

**Affiliations:** 1 College of Information Science & Electronic Engineering, Zhejiang University, Hangzhou 310027, China; 11931084@zju.edu.cn; 2 Xidian Hangzhou Institute of Technology, Xidian University, Hangzhou 311200, China; zyliu402@stu.xidian.edu.cn; 3 Xidian Guangzhou Institute of Technology, Xidian University, Guangzhou 510555, China

**Keywords:** internet of vehicles, action recognition, broad learning, graph feature representation

## Abstract

Recognizing dangerous driving actions is critical for improving road safety in modern transportation systems. Traditional Driver Monitoring Systems (DMSs) often face challenges in terms of lightweight design, real-time performance, and robustness, especially when deployed on resource-constrained embedded devices. This paper proposes a novel method based on 3D skeletal data, combining Graph Spatio-Temporal Feature Representation (GSFR) with a Broad Learning System (BLS) to overcome these challenges. The GSFR method dynamically selects the most relevant keypoints from 3D skeletal data, improving robustness and reducing computational complexity by focusing on essential driver movements. The BLS model, optimized with sparse feature selection and Principal Component Analysis (PCA), ensures efficient processing and real-time performance. Additionally, a dual smoothing strategy, consisting of sliding window smoothing and an Exponential Moving Average (EMA), stabilizes predictions and reduces sensitivity to noise. Extensive experiments on multiple public datasets demonstrate that the GSFR-BLS model outperforms existing methods in terms of accuracy, efficiency, and robustness, making it a suitable candidate for practical deployment in embedded DMS applications.

## 1. Introduction

In recent years, Driver Monitoring Systems (DMSs) have become a critical component in ensuring road safety and reducing traffic accidents. DMSs typically monitor driver behavior to detect dangerous actions, indicative of states such as fatigue, distraction, or impairment, preventing potential traffic incidents. These systems have evolved from traditional methods based on visual image data to more advanced techniques utilizing skeletal data, which provide unique advantages in terms of computational efficiency and robustness. According to the National Highway Traffic Safety Administration (NHTSA), distracted driving alone causes more than 3000 fatal crashes each year, underscoring the need for effective DMS technologies to enhance road safety [1,2,3]. As the reliance on advanced sensor technologies grows, DMSs must operate efficiently under resource constraints, making them a vital aspect of modern transportation infrastructure [4,5,6].

Existing DMS algorithms can be broadly categorized into two approaches: direct image-based detection methods and skeletal data-based detection methods. Direct image-based methods typically rely on convolutional neural networks (CNNs) [7,8] and recurrent neural networks (RNNs) [9,10] or other deep learning models that process raw pixel data to identify dangerous driving behaviors. However, these models often require significant computational resources, which can hinder real-time performance on resource-constrained embedded devices [11,12]. In contrast, skeletal data-based methods focus on analyzing the 3D skeletal features of the driver, providing several advantages over traditional image-based approaches. Skeletal data are computationally less intensive, offer better light invariance, and are more robust to occlusions and noise. These characteristics make skeletal data particularly suitable for deployment on embedded systems with limited resources.

Despite the critical role DMSs play in preventing dangerous driving, existing skeletal data-based algorithms face several challenges in real-world applications. First, they must operate efficiently on embedded devices with limited computational power and storage capacity, necessitating lightweight algorithms that strike a balance between accuracy and efficiency [13]. Additionally, traffic incidents can occur suddenly, requiring real-time detection and immediate response [14]. Furthermore, DMSs must maintain high accuracy in dynamic environments where sensor data can be noisy, incomplete, or missing due to sensor malfunctions or communication issues [15,16].

To address these challenges, this paper proposes a novel, lightweight, real-time, and robust DMS for dangerous driving behavior detection that integrates graph-based approaches with a Broad Learning System (BLS). The core of our method lies in its ability to process 3D skeletal data for dynamic driver behavior analysis, capturing key spatio-temporal features that are crucial for detecting dangerous driving actions. Unlike traditional methods that heavily rely on visual data, our approach focuses on skeletal data, which are not only more efficient in terms of computational requirements but also more robust to occlusions, lighting variations, and noise. The key contributions of this paper are as follows:

1. A Graph Spatio-Temporal Feature Representation (GSFR) method that selects the most relevant keypoints from 3D skeletal data by analyzing spatio-temporal dynamics. This approach improves detection accuracy while reducing computational complexity, ensuring real-time performance on embedded systems. The dynamic selection mechanism also enhances robustness against noisy or missing data.
2. The integration of a Broad Learning System (BLS), a lightweight neural network architecture, to process the selected features efficiently. By using sparse feature selection and Principal Component Analysis (PCA), we reduce the dimensionality of the feature space while retaining essential information, further enhancing real-time detection capabilities.
3. A dual smoothing strategy applied to the BLS output to stabilize predictions over time. This approach combines sliding window smoothing and an Exponential Moving Average (EMA), reducing sensitivity to short-term fluctuations and enhancing the reliability of detection in dynamic driving conditions.
4. Extensive experimental validation on multiple public datasets, demonstrating that the GSFR-BLS model outperforms existing methods in terms of accuracy, real-time performance, and robustness, making it suitable for deployment in real-world DMS applications.

The rest of this paper is organized as follows: Section 2 reviews related work on driver behavior recognition and DMSs. Section 3 describes the proposed GSFR-BLS method in detail. Section 4 presents the experimental setup and results. Finally, Section 5 concludes this paper and suggests potential directions for future research.

## 2. Related Works

### 2.1. Driver Monitoring Systems

Driver Monitoring Systems are critical technologies in intelligent transportation systems, playing a vital role in preventing traffic accidents and enhancing road safety. The DMS primarily monitors driver attention and behavior in real time to detect unsafe driving behaviors indicative of states such as fatigue or distraction. To improve the performance of DMSs, deep learning and pattern recognition methods have been widely applied in these systems [1,2]. Malik et al. proposed an automatic classification system based on driving patterns, utilizing deep learning for driver behavior analysis and recognition, effectively enhancing driving safety [2]. Gupta et al. discussed the potential of deep learning for object detection and scene perception in autonomous driving monitoring and identified future challenges [1]. Fink et al. explored the applications of deep learning in health management monitoring, highlighting its significant advantages in predictive maintenance [3]. These studies demonstrate the prospects of deep learning in DMSs for driver behavior detection and state monitoring. Reddy et al. focused on real-time driver monitoring systems, implementing model compression techniques to enable deep neural networks to achieve real-time driver drowsiness detection on embedded systems [13]. Additionally, Huval et al. validated the reliability of deep learning for driving behavior detection and prediction through highway driving data, further illustrating the applicability of deep learning in various driving scenarios [14]. In the area of multi-sensor information fusion, Qiu et al. discussed the integration of multiple sensor data to meet the needs of behavior recognition in complex driving environments, which is crucial for improving the accuracy of DMSs [15]. Behrendt et al. introduced a deep learning method for traffic light detection and classification, enhancing DMS performance in specific scenarios [16].

Overall, existing research highlights the significant potential of deep learning in the DMS domain, although challenges remain in terms of real-time performance, computational demands, and sensor data integration [1,2,3,13,14,15,16].

### 2.2. Skeleton-Based Action Recognition

Skeleton-based action recognition has become a prominent approach for human action recognition due to its efficiency in capturing spatial and temporal features from skeleton data. Unlike pixel-based methods, skeleton-based models focus on the coordinates of key body joints, which makes them less susceptible to variations in appearance and lighting. Recently, deep learning methods, especially graph convolutional networks (GCNs) and Temporal Convolutional Networks (TCNs), have been widely adopted to model skeleton data for action recognition tasks [7,8]. Yan et al. proposed a Spatial Temporal Graph Convolutional Network (ST-GCN) which captures both spatial and temporal dependencies among joints, setting a foundational approach for skeleton-based action recognition [8]. Shi et al. further extended this approach by incorporating directed graph neural networks to enhance the interpretability and performance of action recognition systems [7]. Another approach proposed by Duan et al. [17] revisits the structure of ST-GCNs and uses optimization strategies that significantly improve recognition accuracy on standard datasets. Progressing from GCNs, CNN-based methods such as the one proposed by Wang et al. have demonstrated efficacy in fusing temporal features through multi-scale convolution operations, which capture motion dynamics effectively [18]. Additionally, RNN-based architectures like those presented by Liu et al. leverage Long Short-Term Memory (LSTM) networks to handle sequential skeleton data, proving effective in modeling temporal dependencies for complex actions [9]. Recent innovations also include Transformer architectures applied to skeleton-based recognition. Xin et al. conducted a comprehensive review on the use of Transformers, emphasizing their ability to capture long-term dependencies more effectively than conventional methods [10]. Furthermore, Su et al. proposed an unsupervised clustering approach for skeleton-based action recognition, facilitating the model’s adaptability to new datasets without extensive labeled data [19].

These advancements collectively highlight the evolution and potential of deep learning techniques in skeleton-based action recognition. However, challenges such as handling noisy data and improving real-time efficiency remain areas for further exploration [20,21].

### 2.3. Broad Learning System

Broad Learning Systems have recently emerged as an efficient alternative to deep learning models, particularly suited for high-dimensional data processing in resource-constrained environments. Unlike conventional deep networks that stack multiple layers, the BLS leverages a single-layer structure to expand feature nodes and enhancement nodes, achieving similar performance to deep networks while significantly reducing training time and computational complexity [22]. Chen and Liu introduced the foundational architecture of the BLS, emphasizing its universal approximation capability and structural simplicity, which makes it ideal for tasks requiring rapid and incremental learning [22]. Following this, Feng and Chen further explored the BLS architecture for neuro-fuzzy systems, applying it to regression and classification tasks with promising results [11]. Peng et al. demonstrated the use of the BLS for emotion recognition using EEG signals, showcasing its versatility in real-time applications [12]. Yu and Hatcher surveyed the broader applications of BLSs, emphasizing their adaptability across various domains [23]. In the healthcare domain, Esteva et al. highlighted BLSs as a powerful tool for medical imaging and diagnostic prediction, facilitating faster model deployment in clinical environments [24]. Lavin et al. developed an advanced version of the BLS for privacy-preserving applications in federated learning systems, demonstrating its potential in distributed settings [25]. Moreover, Zhang and Wang proposed an enhanced BLS variant optimized for predictive maintenance, reducing data dependencies and improving fault detection accuracy [26]. Recent advancements by Li et al. involved a BLS in cybersecurity, employing its rapid training capabilities to detect real-time anomalies in network traffic, further underscoring the BLS’s potential across diverse sectors [27].

These studies collectively underscore the advantages of BLSs as an efficient, scalable, and adaptable alternative to deep learning architectures, particularly in scenarios requiring low-latency model updates and real-time processing [11,12,22,23,24,25,26,27].

## 3. Technical Design of GSFR-BLS

**Figure 1:**
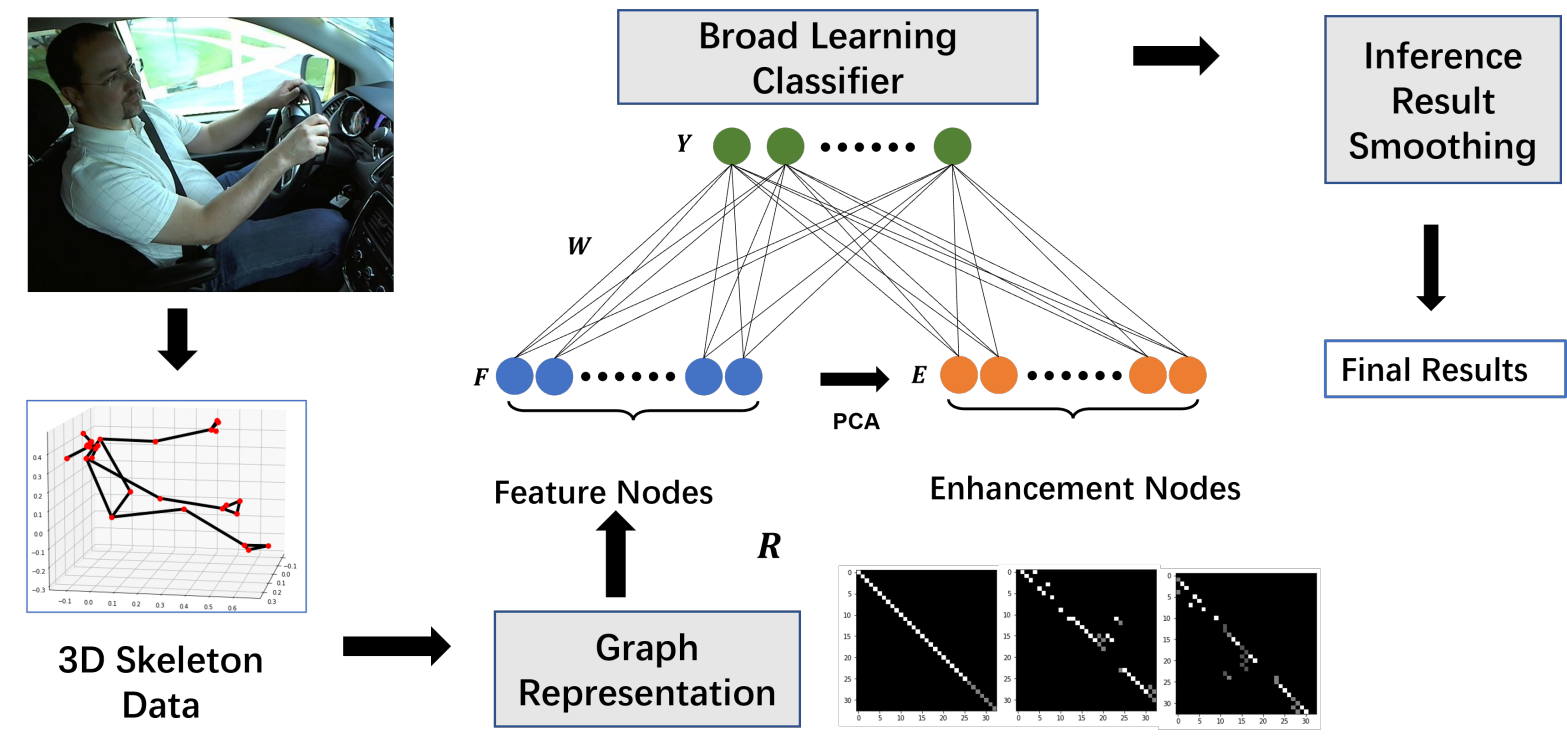
The architecture of the proposed method.

Figure 1 presents the overall procedure for dangerous driving action recognition, which integrates graph representation to dynamically select key skeletal points and construct a spatio-temporal feature graph, enhancing sensitivity to driver movements. Then, the broad learning system with sparse feature selection and PCA is employed to efficiently extract and classify features while reducing computational complexity. To ensure stable and reliable predictions, a dual smoothing strategy combining Sliding Window Smoothing and EMA is applied. This framework enables real-time, lightweight deployment on embedded devices while maintaining high accuracy and robustness in detecting dangerous driving behaviors.

### 3.1. Graph-Based Skeleton Feature Representation

**Figure 2:**
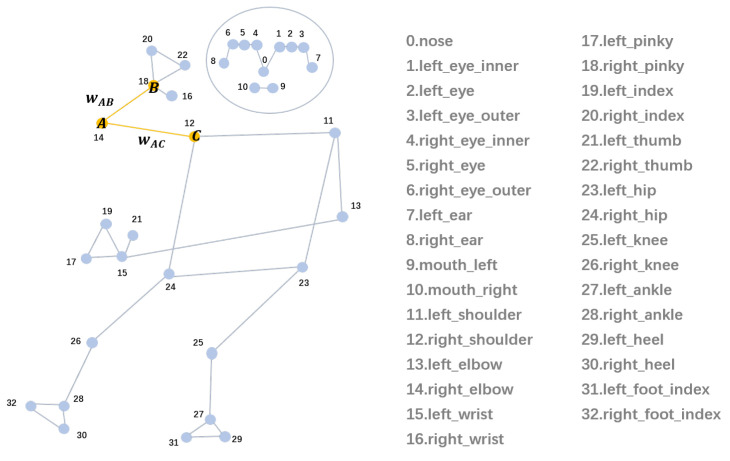
The 33 3D keypoints skeleton model defined by BlazePose.

Figure 2 shows the 33 3D keypoints skeleton model defined by BlazePose [28]. The skeleton model can be represented by a graph structure, where each keypoint can be defined as a vertex, and the connections between keypoints are defined as edges. Therefore, the skeleton keypoints of a frame can be defined as an undirected graph G=(V,E).

In this study, the GSFR algorithm is improved by incorporating spatio-temporal dynamic selection. Unlike the traditional GSFR that processes static adjacency matrices, a dynamic mechanism that selects the most relevant keypoints by analyzing the movement of each point over multiple frames is introduced. This allows the system to focus on the keypoints that exhibit significant movement, which is more indicative of dangerous driving behaviors.

For each frame, the skeleton data consist of the 3D positions of keypoints, denoted as $P_{i}^{t}=\left(x_{i}^{t},y_{i}^{t},z_{i}^{t}\right)$, where *i* represents the keypoint index and *t* represents the frame number. To capture the temporal dynamics, the displacement of each keypoint between consecutive frames is computed. The displacement of keypoint $P_{i}$ between frame *t* and t−1 is defined as

(1) $$\Delta P_{i}^{t}=P_{i}^{t}-P_{i}^{t-1}=\left(x_{i}^{t}-x_{i}^{t-1},y_{i}^{t}-y_{i}^{t-1},z_{i}^{t}-z_{i}^{t-1}\right).$$

The displacements across multiple frames are accumulated to evaluate the significance of each keypoint’s movement over time.

To dynamically select keypoints that are most relevant for action recognition, a thresholding mechanism based on the magnitude of movement is introduced. Instead of using a fixed threshold, the threshold is defined dynamically based on the average movement magnitude of all keypoints in a frame. The accumulated movement of each keypoint over a window of *T* frames is calculated as

(2) $$\Delta P_{i}^{\mathrm{total}}=\sum_{k=1}^{T}\Delta P_{i}^{t-k}.$$

The global average movement of keypoints in frame *t* is defined as

(3) $${\bar{\Delta P}}^{\mathrm{total}}=\frac{1}{N}\sum_{i=1}^{N}\Delta P_{i}^{\mathrm{total}}$$

where *N* is the total number of keypoints. The threshold is dynamically set as a multiple of the global average movement:

(4) $$\theta =\beta \cdot {\bar{\Delta P}}^{\mathrm{total}}$$

where β is a hyperparameter controlling the sensitivity of the threshold. A keypoint is considered significant if its accumulated movement $\Delta P_{i}^{\mathrm{total}}$ exceeds the threshold θ:

(5) $$\mathrm{important\_points}=\{P_{i}|\Delta P_{i}^{\mathrm{total}}>\beta \cdot {\bar{\Delta P}}^{\mathrm{total}}\}.$$

This ensures that only keypoints with significant movement are used to form the dynamic neighborhood, reducing computational overhead by excluding less important points.

After selecting the most relevant keypoints based on their movement over time, the representation matrix *R*, by combining the selected keypoints’ information and the adjacency relationships between them, is constructed.

The adjacency matrix *A* is split into two parts: the out-degree matrix $A_{1}$ and the in-degree matrix $A_{2}$. Both matrices are filtered using a keypoint importance matrix *K*, which reduces the contribution of less significant keypoints (such as those in the lower body). This decomposition allows us to separately account for the spatial influence of incoming and outgoing connections between keypoints. After filtering and normalization, the representation matrix *R* is constructed by concatenating the following matrices:

(6) $$R=[I^{\prime },A_{1}^{\prime },A_{2}^{\prime }]$$

where $I^{\prime }$ represents the identity matrix filtered by keypoint importance, capturing the self-information of each keypoint. $A_{1}^{\prime }$ and $A_{2}^{\prime }$ represent the normalized out-degree and in-degree matrices, capturing the relationships between keypoints based on their spatial and temporal dynamics.

The representation matrix *R* captures the selected keypoints’ self-information and their adjacency relationships, and this matrix is used as the input to the BLS for further classification. The spatial-temporal information embedded in *R* enables the BLS to capture nuanced movement patterns indicative of dangerous driving actions.

The procedure for constructing the representation matrix *R* in Algorithm 1 dynamically selects key skeletal points based on motion displacement, applies a threshold from the global average movement to filter irrelevant points, updates adjacency matrices, and finally concatenates the normalized identity and adjacency matrices to serve as input to the BLS for efficient and robust feature extraction in dangerous driving action recognition.
**Algorithm 1:** Constructing representation matrix *R* with spatio-temporal dynamic selection.
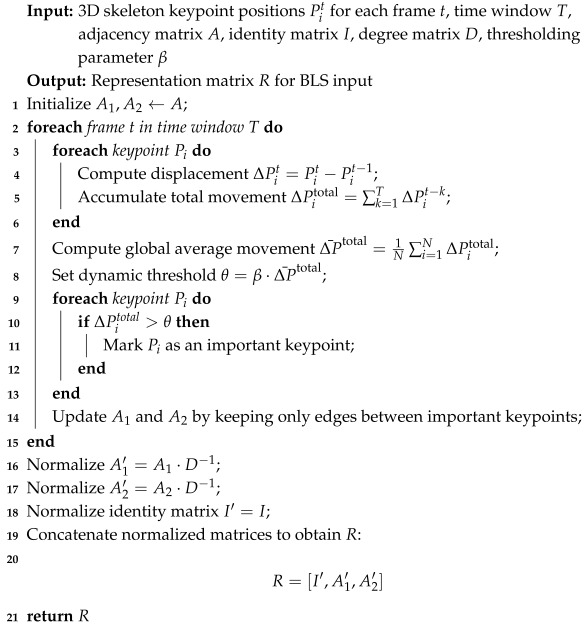


### 3.2. Broad Learning Classifier with Enhanced Feature Mapping

The representation matrix *R*, constructed from the spatio-temporal dynamic selection of keypoints, serves as the input to the broad learning classifier. *R* contains the fused information of the keypoints’ spatial and temporal relationships, and it is passed through the feature mapping layer and enhanced feature mapping layer for classification.

The feature mapping layer takes the matrix *R* as input and transforms it into the feature nodes. By transforming *R* into feature nodes, the essential spatial-temporal features across keypoints are captured. This is achieved by performing linear mapping on *R* through randomly initialized weights and biases. The process is formulated as

(7) $$F_{i}=R\cdot W_{Fi}+\beta_{Fi},\;i=1,\ldots ,N_{2}$$

where $F=[F_{1},\ldots ,F_{N_{2}}]$ represents the set of feature nodes, each corresponding to a different feature window. Furthermore, $W_{Fi}$ and $\beta_{Fi}$ are the random weights and biases for the transformation.

This step generates $N_{2}$ windows, each containing $N_{1}$ feature nodes. No iterative training is required at this stage, and the transformation is efficient.

A combination of sparse feature selection and PCA is proposed, operating on the output from the feature mapping layer *F*. These methods ensure that only the most relevant features from the matrix *R* are retained, leading to improved model efficiency and better classification performance.

1. Sparse feature selection: The L1 regularization to the feature nodes to enforce sparsity and remove noisy or redundant features is applied. The objective function for sparse feature selection is defined as

(8) $$\min_{W}{∥F\cdot W-X∥}_{2}^{2}+\lambda {∥W∥}_{1}$$

where *F* is the feature node matrix obtained from the previous layer, *W* are the learned sparse weights, and λ is the regularization parameter controlling the sparsity.

2. Principal Component Analysis: After sparse feature selection, the PCA on the selected features to reduce their dimensionality further is performed. This ensures that the most informative features are preserved while simultaneously lowering computational complexity:

(9) $$E_{j}=\mathrm{PCA}\left(F\right)\cdot W_{Ej}+\beta_{Ej},\;j=1,\ldots ,N_{3}$$

where $E=[E_{1},\ldots ,E_{N_{3}}]$ represents the enhanced nodes, and $W_{Ej}$ and $\beta_{Ej}$ are the random weights and biases for the enhancement mapping.

The final output *Y* is obtained by concatenating the feature nodes *F* and enhanced nodes *E* and applying a ridge regression model to learn the weights *W* that map these nodes to the target output *Y*:

(10) Y=[F|E]W

where [F|E] is the concatenation of the feature and enhanced nodes, and *W* represents the learned weights used to map the features to the output labels.

The learned weights *W* are obtained using the Moore–Penrose pseudoinverse:

(11) $$W={\left[g\left(F|E\right)\right]}^{+}Y$$

where g(x) denotes the activation function applied to the feature and enhanced nodes, and ${\left[g\left(F|E\right)\right]}^{+}$ is the pseudoinverse of the transformed feature space.

During inference, the matrix *R* is passed through the same feature mapping layer and enhanced feature mapping layer. The saved weights *W* are then used to compute the classification result, providing real-time predictions with high accuracy.

The broad learning classifier (BLC) produces an output matrix *Y* based on the input representation matrix *R*. This output o(t) represents the raw classification probabilities, which will undergo a smoothing process to stabilize the predictions. The final classification result o(t) at frame *t* is computed as

(12) $$o\left(t\right)=\mathrm{softmax}\left(Y_{t}\right)$$

where $Y_{t}$ is the output from the BLS at frame *t*, and the softmax function converts the output logits into classification probabilities for the respective classes.

The raw classification output o(t) is then passed into the inference result smoothing process to improve robustness and reduce false positives.

### 3.3. Inference Result Smoothing for Robust Classification

To further enhance the robustness of the classification process, a two-stage smoothing strategy is applied to the raw classification results from the BLS. The raw classification output o(t) from the BLS, representing the predicted probabilities for each class, is smoothed using a combination of sliding window smoothing and EMA to reduce short-term fluctuations and maintain sensitivity to long-term trends.

The first step is to apply a sliding window over the BLS classification results to reduce short-term fluctuations. This involves averaging the classification outputs over a window of consecutive frames. The sliding window-smoothed classification result is calculated as follows:

(13) $$o^{\mathrm{smoothed}}\left(t\right)=\frac{1}{w}\sum_{j=t-w+1}^{t}o\left(j\right)$$

where o(j) represents the classification output at frame *j*, *w* is the size of the sliding window, and $o^{\mathrm{smoothed}}\left(t\right)$ is the smoothed classification output at frame *t*.

After applying sliding window smoothing, the classification results are further smoothed using EMA. This step ensures that the classification output responds to long-term trends while reducing jitter. The EMA-smoothed classification result is computed as

(14) $$o^{\mathrm{EMA}}\left(t\right)=\gamma \cdot o^{\mathrm{smoothed}}\left(t\right)+\left(1-\gamma \right)\cdot o^{\mathrm{EMA}}\left(t-1\right)$$

where $o^{\mathrm{EMA}}\left(t\right)$ is the exponentially smoothed classification result at frame *t*, γ is the smoothing factor, and $o^{\mathrm{EMA}}\left(t-1\right)$ is the EMA result from the previous frame.

The final classification result after the dual smoothing process is given by

(15) $$o^{\mathrm{final}}\left(t\right)=o^{\mathrm{EMA}}\left(t\right).$$

This final smoothed classification output $o^{\mathrm{final}}\left(t\right)$ reduces false positives and stabilizes the BLS predictions, making the classification more robust in real-world applications. This dual-smoothing approach effectively reduces noise in the classification outputs, improving robustness against false positives.

Most parameters in Equations (1)–(15) are determined dynamically during data processing and model training, reducing the need for manual tuning and enhancing robustness across diverse scenarios. However, certain key parameters require explicit initialization, including the time window (*T*), dynamic threshold multiplier (β), regularization strength (λ), sliding window size (*w*), and smoothing factor (γ). The time window is set to 60 frames (approximately 2 s) as suggested by [29], aligning with typical human action durations in behavior recognition tasks. The dynamic threshold multiplier (β) is set to 2.0 based on the 3σ principle [30], effectively filtering noise while retaining relevant data. The regularization strength (λ) is set to 0.01 following [31] to prevent overfitting, while the sliding window size (*w*) of 10 frames and smoothing factor (γ) of 0.8 are chosen based on [32,33], respectively, to ensure stable and noise-resistant temporal feature extraction. These parameter settings balance computational efficiency and accuracy, providing a reliable foundation for our approach.

## 4. Experiments

### 4.1. Dataset

The State Farm dataset, featured in the “State Farm Distracted Driver Detection” competition on Kaggle [34,35,36], is used in this study. Additionally, the Driver Skeleton [37,38] is a video dataset from which each frame is extracted to create an image dataset. This dataset, collected through dashboard cameras, is divided into 10 categories identified by c0, …, c9, as shown in Table 1. The State Farm dataset is a widely used dataset for distracted driving behavior analysis. It contains a collection of images and videos from in-car cameras, which capture various driving scenarios. The dataset is labeled with different driving behaviors, such as whether the driver is engaged in distracted driving or focused on the road. The State Farm dataset is commonly used for training and evaluating models that detect distracted driving by analyzing visual cues such as facial expressions, head movements, and hand positions. This dataset serves as a benchmark for driver behavior classification tasks and provides valuable data for studying driver attentiveness in various road conditions. The Driver Skeleton dataset is designed for studying driver behavior recognition using 3D skeletal data. This dataset focuses on capturing the body keypoints of the driver using skeletal tracking technology, which enables the analysis of subtle movements such as hand gestures, head orientation, and body posture. The dataset provides 3D coordinates of body keypoints (e.g., joints and limbs) and can be used to recognize and classify different driving behaviors, including potentially dangerous actions. The main advantage of using skeletal data over traditional image data is their robustness to lighting variations and occlusions, making them more suitable for real-time monitoring and behavior classification.

**Figure 3:**
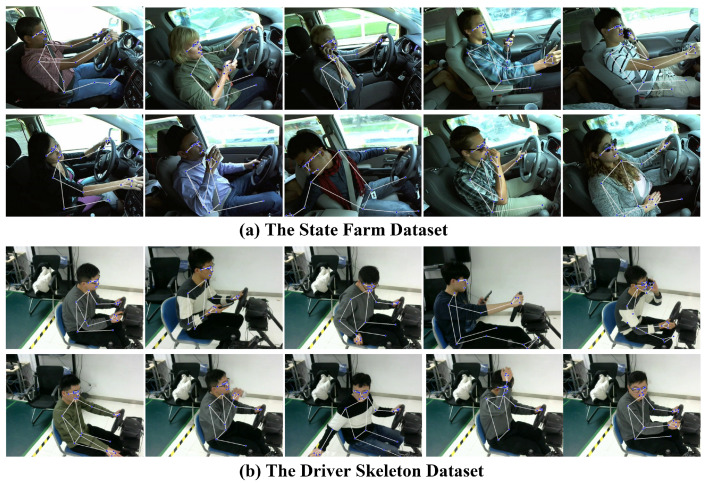
Skeleton representation in datasets.

BlazePose is used to obtain (33, 3) skeleton keypoints data for each image in the State Farm dataset and is saved to a CSV file. Because BlazePose is not always able to accurately estimate skeleton keypoints, data cleaning is performed to remove outliers from each category using kNN. The scheme of applying kNN for data cleaning is based on the assumption that similar data points should be close to each other in the feature space. Therefore, data points significantly deviating from this neighborhood are considered abnormal and removed from the dataset to improve data quality. By removing outliers, each category exhibits significant differences in distance within the feature space, beneficial for training classification models. During data cleaning, all data of the same category are inputted. When processing a specific data point, the algorithm uses the maximum difference between the keypoint coordinates of the data and all other data as the distance measure, finding *k* nearest neighbors for each data point. If its annotation category differs from its neighbors, it is considered an outlier and removed. Figure 3 visualizes skeleton keypoints and links on the original image. The number of images in each category is shown in Table 1. The official data partitioning scheme of the State Farm dataset is followed, and the standard 80% and 20% split strategy is applied to both datasets to ensure a rigorous evaluation [34,39,40]. These datasets consist of diverse driving behaviors performed by different drivers or simulated drivers, which helps mitigate the risk of overfitting to a single individual’s movement patterns.

### 4.2. Evaluation Indicators

The following indicators are used for evaluating the driving action recognition algorithm:

- Accuracy (Acc): It evaluates the accuracy of model classification, referring to the accuracy of the testing set in this study.
- Confusion matrix (CM): It represents the actual category and predicted category as a two-dimensional matrix, with each element showing the combination of actual and predicted categories.
- Parameters (Param): To consider the minimum number of parameters required to support model inference in practical applications, the size of the saved parameter model file as an indicator is utilized. Comparing the number of parameters with many machine learning models helps evaluate whether the model is lightweight. A smaller number of parameters signifies that the model requires less memory and computation, making it more lightweight.
- Inference time (IT): This is the time taken to infer the testing set, visually evaluating the real-time performance of model inference.

### 4.3. Experiment Environment

In this study, GSFR ablation experiments and the driving action recognition method comparative experiments are conducted using Python 3.10 on a Windows 10 desktop computer equipped with an i7-8700 3.20 GHz CPU and 24 GB 2666 MHz memory. The deployment experiment is conducted on an NVIDIA Jetson TX2, an embedded device designed for deep learning. However, the CPU is employed for the experiment. The CPU comprises ARM v8 64-bit CPU clusters, including HMP Dual Denver 2/2 MB L2 and Quad ARM® A57/2 MB L2.

### 4.4. GSFR Ablation Experiment

To verify the effectiveness of GSFR, the difference between the presence and absence of GSFR processes is tested on the original BLS, deep neural network (DNN), and GCN. The values are set as $N_{1}=15$, $N_{2}=10$, and $N_{3}=500$. These values correspond to the number of feature nodes in each window (15 nodes per window with a total of 10 windows) and the number of enhancement nodes (500 nodes). The model settings of the BLS are as follows: the number of feature nodes in each window is 15, with 10 windows. The number of enhancement nodes is 500. The hidden layers of the DNN and GCN consist of three layers, with 64, 128, and 64 neurons, respectively. The dropout probability is 0.2. The Adam optimizer with $beta_{1}$ = 0.9 and $beta_{2}$ = 0.999 is used, with a learning rate of 0.001 and a learning rate decay of 0.001. The model is trained for 100 epochs. Table 2 demonstrates the improvement of GSFR on classifier performance. Whether utilizing a BLS or DNN, employing GSFR significantly improves accuracy.

**Figure 4:**
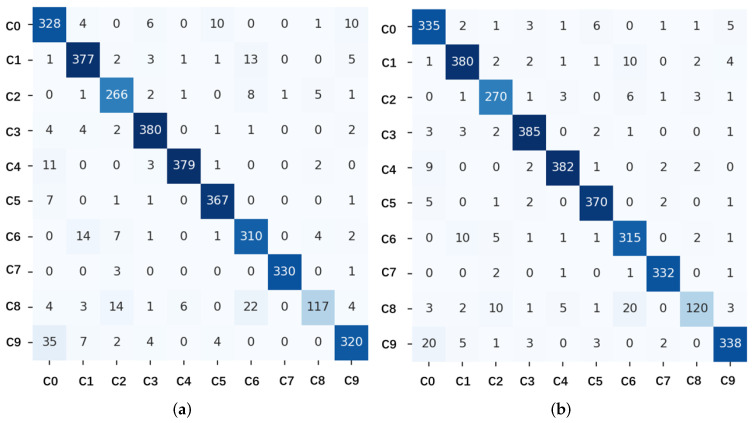
The confusion matrices without GSFR of BLS on the Driver Skeleton dataset. (**a**) CM of BLS without GSFR. (**b**) CM of BLS with GSFR.

Figure 4 shows the confusion matrices of the BLS without and with GSFR. From the confusion matrices, it can be seen that the number of correct classifications in most categories has increased after using GSFR. However, the model is prone to confusion between c0 to c9, meaning the data for safe driving and chatting with passengers are relatively similar. In the skeleton coordinate data, only the facial keypoints are slightly offset, while other keypoints remain almost the same. Distinguishing these two actions is challenging. The GSFR-BLS model demonstrates consistent improvements in precision and recall across most categories, especially for challenging ones like c8 (reaching behind) and c0 (safe driving). These enhancements highlight GSFR’s ability to dynamically focus on relevant skeletal features, improving discrimination of overlapping or subtle behaviors. The overall accuracy improvement from 93.30% to 93.64% underscores the added value of GSFR-BLS, making it a more robust choice for driver behavior monitoring. The model exhibits significant differences in performance across various driving behavior categories. High-performance categories include c1 (texting—right), c3 (texting—left), c5 (operating the radio), and c7 (hair and makeup), where both precision and recall exceed 95%, demonstrating the model’s superior ability to recognize behaviors with prominent skeletal dynamics. These actions often involve distinct hand or head movements, which are easily captured by the skeletal extraction module. However, for low-performance categories like c8 (reaching behind) and c0 (safe driving), the model faces considerable challenges. For instance, c8 achieves precision and recall of 79.1% and 76.2%, respectively, due to its subtle behavioral features, which are often confused with c7 (hair and makeup). Similarly, c0 achieves a recall of 94.6%, but its precision is only 89.1%, mainly due to overlapping dynamic features with c9 (talking to passenger). These results indicate that while the model excels in capturing distinct behaviors, it remains limited in distinguishing between behaviors with similar or less pronounced dynamics.

### 4.5. Classification Experiment

**Figure 5:**
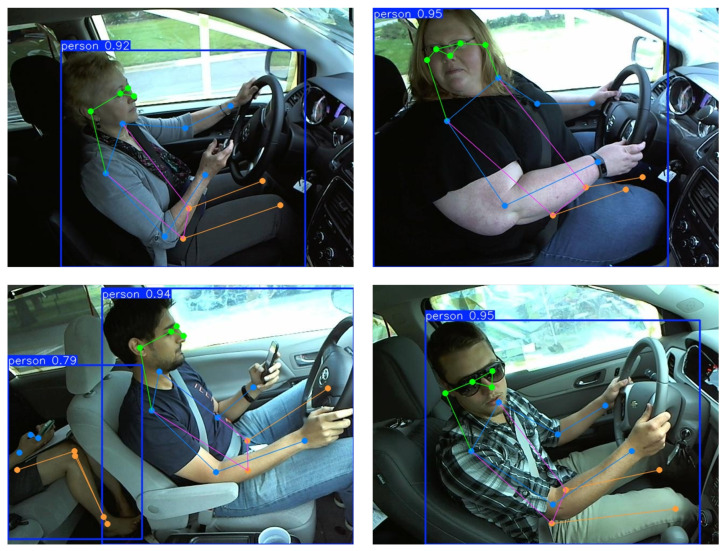
The visualization of driving action recognition results on the State Farm dataset.

The visualized results demonstrate that the proposed method effectively extracts skeletal features from drivers of different body types and genders, providing robust support for behavior analysis, as shown in Figure 5. The method accurately captures key skeletal nodes and their dynamic distributions, including head (green nodes and lines); shoulders, arms, and hands (blue nodes and lines); torso (purple nodes and lines); and lower limbs (orange nodes and lines). These features are consistently extracted across diverse scenarios, such as smaller or larger body types, male and female drivers, and varying postures. For instance, in cases like texting with one hand, talking on the phone, or safe driving, the system clearly distinguishes between behaviors based on the spatial and temporal relationships of skeletal keypoints. Additionally, even in complex environments with multiple individuals, the method isolates the driver’s skeletal features, ensuring accurate representation. The adaptability and robustness of the skeletal extraction process provide high-quality inputs for driving behavior classification, establishing its suitability for diverse and real-world applications.

The accuracy, inference time, and parameters of the proposed driving action recognition method are compared with those of other mainstream classifiers: machine learning classifiers, such as random forests [41], gradient boosting [42], and support vector machine (SVM) [43]; deep learning classifiers, including DNN, 1D-CNN, and GCN, which have identical hidden layer structures consisting of layers with 64, 128, and 64 neurons, respectively; and the original BLS. The hyperparameters of the machine learning classifiers mentioned above are the default parameters in Python’s sklearn library.

Table 3 demonstrates the excellent performance of the proposed driving action recognition method, which offers high accuracy, short inference time, and lightweight characteristics across different datasets. Typically, machine learning classifiers require large parameters to achieve high accuracy. Deep learning classifiers often struggle to achieve high accuracy due to a lack of efficient feature representation, and their complex computing processes result in significant inference time. Overall, the original BLS has comprehensive advantages, and the proposed method improves the classification accuracy of the original BLS without incurring significant costs. To validate the effectiveness of the proposed method, comparisons are conducted with several recent state-of-the-art approaches relevant to Driver Monitoring Systems (DMSs). On the State Farm dataset, the proposed method was compared with SC-GCN [39] and DDDS [34]. These models are widely recognized in the DMS domain for driver action recognition but are not optimized for lightweight performance. On the Driver Skeleton dataset, the proposed method was evaluated against 2S-GCN-TAtt [38] and Beh-MSFNet [37]. Although Beh-MSFNet achieves slightly higher accuracy, its parameter size (3614.72 KB) is significantly larger compared to the proposed method (507 KB), highlighting the advantage of the GSFR-BLS in achieving competitive accuracy while maintaining a compact model size. This balance between accuracy and model efficiency makes the proposed method particularly suitable for deployment in resource-constrained environments.

**Figure 6:**
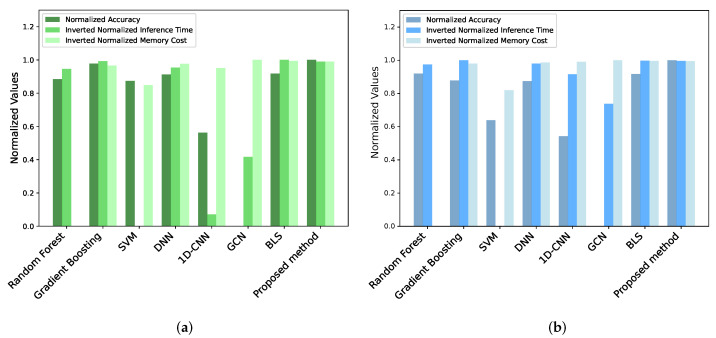
The classification performance comparison on State Farm dataset and Driver Skeleton dataset. (**a**) Performance comparison on State Farm dataset. (**b**) Performance comparison on Driver Skeleton dataset.

Figure 6 evaluates the capabilities of the above classifiers based on accuracy, inference time, and parameters. To accommodate the significant numerical gaps between different indicators, the Min-Max Normalization is applied as in (16) to each indicator across various models, constraining their values to a range of (0, 1). Shorter inference time and smaller parameter costs indicate better performance. Therefore, the values for the normalized inference time and normalized parameters have been inverted. This approach enables a more intuitive evaluation of individual indicators within the compared models and provides a measure of the model’s overall capabilities. Figure 6 illustrates that the proposed driving action recognition method performs well across all indicators despite not being the best in any single metric.

(16) $$x_{normalization}=\frac{x-Min}{Max-Min}$$

### 4.6. Deployment Experiment

To verify the actual deployment performance of the workflow, the workflow is tested on an NVIDIA Jetson TX2. Inference is performed using only the CPU of the TX2, not the GPU. The proposed workflow is observed for real-time performance using only the CPU with ARM architecture.

**Figure 7:**
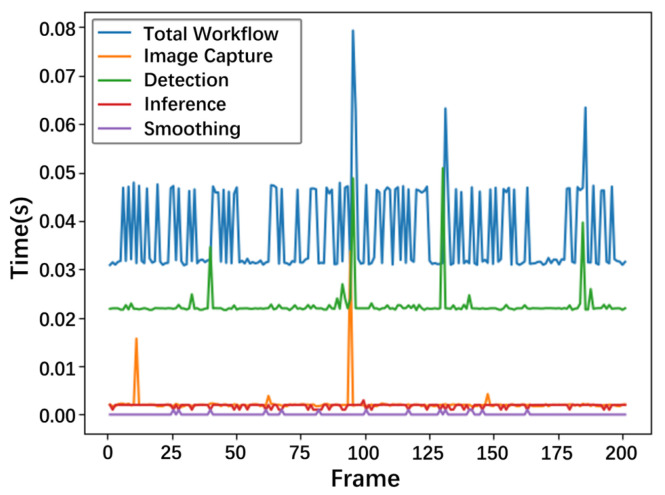
The time-cost statistics of modules in the workflow.

Figure 7 visualizes 200 frames of each module’s time cost in the workflow during runtime. The average data of statistical information are shown in Table 4. They manifest the low time cost. Most of the data fluctuations in Figure 7 come from the time it takes to read a single-frame image from the camera. The deployment performance can reach 26.67 FPS or above, which provides real-time performance for the dangerous driving action recognition task.

**Figure 8:**
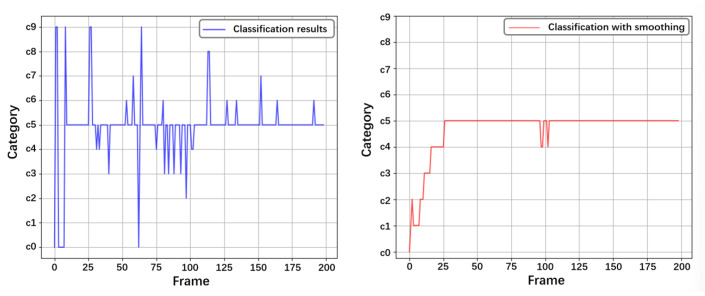
Noise reduction performance of smoothing.

In addition, the smoothing effect of the smoothing algorithm on the results is visualized to verify its effectiveness in noise reduction. The window size of the smoothing algorithm is set to 20, which means that the current detection result will also consider the detection results of the first 20 frames. The detection results are shown in Figure 8, and it can be seen that the smoothing algorithm significantly reduces the noise in the detection results. Combined with historical data, it provides more stable and reasonable detection results for coherent human movements.

## 5. Conclusions

This study presents a novel, lightweight, and real-time method for dangerous driving action recognition based on 3D skeletal data, specifically designed for deployment in resource-constrained embedded devices within a DMS. The proposed approach leverages GSFR to dynamically select relevant 3D skeletal keypoints, enhancing feature representation while reducing computational complexity and improving robustness to noisy or incomplete data. Additionally, the BLS, integrated with sparse feature selection and PCA, optimizes computational efficiency and supports real-time processing. A dual smoothing strategy, combining sliding window smoothing and EMA, stabilizes the classification outputs, making the system more reliable in dynamic driving conditions.

Experimental validation on several public datasets, including the State Farm and Driver Skeleton datasets, demonstrates that the GSFR-BLS model significantly outperforms existing methods in terms of accuracy, efficiency, and robustness, highlighting its suitability for practical DMS applications. However, the model’s performance is still influenced by the accuracy of human pose estimation and potential data imbalance issues. Future work will focus on incorporating raw image data alongside skeletal information to improve classification accuracy and address challenges in real-world applications, further enhancing the system’s robustness in dynamic environments. Future work will aim to extend the system’s applicability by collecting data from more diverse driving scenarios, including real-world environments with a broader range of driver demographics and driving conditions (e.g., urban vs. rural settings, varying age groups, and varying experience levels). This will enable testing in complex and dynamic settings to ensure robustness and reliability in safety-critical applications, ultimately improving the model’s performance across a wider range of real-world situations. Future advancements in DMS applications should be anticipated to be deeply integrated with Intelligent Transportation Systems. Therefore, it is imperative to explore the development of edge–cloud collaborative DMS technologies and applications involving Vehicle-to-Everything [44] communication and Unmanned Aerial Vehicles [45].

## Figures and Tables

**Table 1:** Classification of State Farm Dataset.

Action Number	Category	State Farm	Driver Skeleton
c0	Safe driving	1860	3928
c1	Texting—right	1932	4118
c2	Talking on the phone—right	1444	4125
c3	Texting—left	1887	4201
c4	Talking on the phone—left	1847	3946
c5	Operating the radio	2011	3609
c6	Drinking	1763	4019
c7	Reaching behind	1608	3753
c8	Hair and makeup	965	3533
c9	Talking to passenger	1826	3860

**Table 2:** The ablation experiment results.

Dataset	Classifier	GSFR	Acc(%)
The State Farm dataset	BLS	✓	93.639 (+1.103)
		✕	92.536
	DNN	✓	93.287 (+1.253)
		✕	92.034
	GCN	✓	73.892 (+3.219)
		✕	70.673
The Driver Skeleton dataset	BLS	✓	93.512 (+1.841)
		✕	91.671
	DNN	✓	92.606 (+1.865)
		✕	90.741
	GCN	✓	74.543 (+3.114)
		✕	71.429

**Table 3:** Comparative experiment with the baseline and the state-of-the-art methods.

Dataset	Classifier	Acc (%)	IT (s)	Param (KB)
State Farm	Random forest	91.372	0.202	44,439
	Gradient boosting	93.564	0.064	1612
	SVM	91.112	3.023	6786
	DNN	92.034	0.179	1128
	1D-CNN	83.834	2.813	2253
	GCN	70.673	1.779	81
	BLS	92.164	0.041	364
	SC-GCN [39]	91.45	/	/
	DDDS [34]	93.12	/	/
	Proposed method	93.639	0.071	507
Driver Skeleton	Random forest	91.738	0.526	77,431
	Gradient boosting	90.830	0.113	1641
	SVM	85.535	16.314	14,098
	DNN	90.741	0.432	1128
	1D-CNN	83.395	1.481	809
	GCN	71.429	4.356	81
	BLS	91.671	0.151	364
	2S-GCN-TAtt [38]	90.5	/	/
	Beh-MSFNet [37]	95.4	/	3614.72
	Proposed method	93.512	0.182	507

**Table 4:** Average time cost of workflow modules.

Modules	Time Cost
Image Capture	0.0022 s
Detection	0.0225 s
Inference	0.0018 s
Smoothing	$8.2133\times 10^{-5}$ s
Total Workflow	0.0375 s

## Data Availability

Data is unavailable due to privacy restrictions.
